# Synthesis and Luminescent Properties of Barium Molybdate Nanoparticles

**DOI:** 10.3390/ma16217025

**Published:** 2023-11-03

**Authors:** Maria Gancheva, Reni Iordanova, Iovka Koseva, Georgi Avdeev, Gergana Burdina, Petar Ivanov

**Affiliations:** 1 Institute of General and Inorganic Chemistry, Bulgarian Academy of Sciences, “Acad. G. Bonchev” Str., bl. 11, 1113 Sofia, Bulgaria; reni@svr.igic.bas.bg (R.I.); ikosseva@svr.igic.bas.bg (I.K.); g.naburdina@abv.bg (G.B.); 2 Institute of Physical Chemistry “Acad. Rostislaw Kaischew”, Bulgarian Academy of Sciences, “Acad. G. Bonchev” Str., bl. 11, 1113 Sofia, Bulgaria; g_avdeev@ipc.bas.bg; 3 Institute of Optical Materials and Technologies “Acad. Jordan Malinowski”, Bulgarian Academy of Sciences, “Acad. G. Bonchev” Str., bl. 109, 1113 Sofia, Bulgaria; petar@iomt.bas.bg

**Keywords:** BaMoO_4_, high-energy ball milling, solid-state reaction, nanoparticles, infrared absorption bands, optical band gaps, blue and green photoluminescence, color coordinates

## Abstract

BaMoO_4_ was obtained via facile mechanochemical synthesis at room temperature and a solid-state reaction. An evaluation of the phase composition and structural and optical properties of BaMoO_4_ was conducted. The influence of different milling speeds on the preparation of BaMoO_4_ was explored. A shorter reaction time for the phase formation of BaMoO_4_ was achieved using a milling speed of 850 rpm. A milling speed of 500 rpm led to partial amorphization of the initial reagents and to prolongation of the synthesis time of up to 3 h of milling time. Solid-state synthesis was performed via heat treatment at 900 °C for 15 h. X-ray diffraction analysis (XRD), infrared (IR) and UV diffuse reflectance (UV-Vis) and photoluminescence (PL) spectroscopy were carried out to characterize the samples. Independently of the method of preparation, the obtained samples had tetragonal symmetry. The average crystallite sizes of all samples, calculated using Scherrer’s formula, were in the range of 240 to 1540 Å. IR spectroscopy showed that more distorted structural MoO_4_ units were formed when the compound was synthesized via a solid-state reaction. The optical band gap energy of the obtained materials was found to decrease from 4.50 to 4.30 eV with increasing crystallite sizes. Green- and blue-light emissions were observed for BaMoO_4_ phases under excitation wavelengths of 330 and 488 nm. It was established that the intensity of the PL peaks depends on two factors: the symmetry of MoO_4_ units and the crystallite sizes.

## 1. Introduction

BaMoO_4_ is a compound that belongs to transition-metal oxides with a scheelite-type structure. This phase is an important material and has useful applications in different scientific and technical areas as photocatalysts [1], anode materials for lithium ion batteries (LIB) and sodium ion batteries (SIB) [2], solid-state lasers [3], phosphors [4,5] and host matrices for doping with Re^3+^ ions [5,6,7]. BaMoO_4_ possesses good thermal and chemical stability. It is a semiconductor material with wide optical band gap and high luminescence at room temperature [1,3,4,5,6,7,8,9]. In the scheelite structure, Ba ions are coordinated with eight oxygen atoms, and Mo ions are connected to four oxygen atoms, forming MoO_4_ groups [10]. The MoO_4_ structural unit plays an important role in the electronic structural order and luminescence properties. J.C. Sczancoski et al. reported that the photoluminescence (PL) emission is adjudicated to the MoO_4_^2−^ complex, where cations like Ca^2+^, Sr^2+^ and Ba^2+^ acts as lattice-modifying agents, affecting the photoluminescence property directly [11]. There are data that provide evidence that BaMoO_4_ exhibits green and/or blue emissions at room temperature depending on its morphology, defects, crystallite size, different excitation sources and method of preparation [3,4,5,6,7,8,9,10,11,12,13,14,15,16,17,18,19,20,21,22,23,24,25,26]. The attention of researchers is focused on fast chemical or physical synthesis of BaMoO_4_ under mild conditions and establishing the relationship between its structure, morphology and luminescence properties [10,20,21,22,23,24,25]. BaMoO_4_ particles with shuttle-like microcrystals and an octahedral form exhibit blue emission at room temperature [15]. According to L. Ma et al., BaMoO_4_ with various morphologies, such as ellipsoid-like, peanut-like, cube-like and flower-like, with sizes above 0.25 μm, exhibited the highest, high, middle and lowest photoluminescent emissions at 560 nm [19]. On the other hand, BaMoO_4_ with nest-like and decahedron particles show strong green emission and weak red emission [16]. Micron-octahedron and micron-flower BaMoO_4_ obtained via the sonochemical method exhibited a strong and broad green emission peak with a maximum at around 508 nm [17]. Y. Wang et al. investigated the influence of defects and Jahn–Teller distorted MoO_4_ symmetry on the shape and position of the blue emission of BaMoO_4_ [20]. The blue emission of BaMoO_4_ powder or film was registered under excitation wavelengths of 250, 280, 350, 360 and 370 nm [9,14,23,25]. Photoluminescence in the green range was visible at the same wavelengths and at higher ones [16,17,21]. According to many authors, the appearance of blue or green emissions strongly depend on the types of defects that form during the experimental conditions and the morphology of the final products [4,12,13,20,22]. Several studies have reported that morphological control can be used to adjust the photoluminescence properties in the crystallite phases with the scheelite-type structure [15,16,17]. In the literature, there are data that show that the PL intensity and position of band emissions depend on the thermal treatment conditions of powders [24,25].

Different physicochemical routes have been applied for the preparation of BaWO_4_, such as solid-state reactions [5], hydrothermal synthesis [2,4,9,18], the aqueous mineralization process [15], coprecipitation [13,21], the sonochemical method [1,17], microwave-assisted synthesis [14,23], sol-gel [6], the complex polymerization method [3,24], laser–induced synthesis [25] and mechanochemical synthesis [27,28,29,30]. For example, the BaMoO_4_ phase obtained through the ball milling of initial mixtures of BaCO_3_ + MoO_3_ and BaCO_3_ + Na_2_MoO_4_ can be used in the NIR pigment applications [27]. X.B. Chen et al. applied the high-temperature ball milling (HTBM) method for the preparation of BaMoO_4_:Eu^3+^ red phosphors [28]. On the other hand, AMoO_4_ (A = Ba, Sr) films were fabricated via a ball-rotation-assisted solid/solution reaction at room temperature [29]. R.C. Lima et al. [31] reported that additional milling led to an increase in the intensity of the emission spectra. Mechanochemical treatment has several advantages, such as shorter reaction time, cost-effectiveness, simplicity and reliability. The mechanochemical activation of solids leads to the appearance of fresh areas and active defects, which improves their catalytic, electrical and optical characteristics. The focus of our group is on the effects of different ball milling conditions for faster synthesis of a variety of inorganic compounds at ambient temperature [32,33,34]. This motivated us to use mechanochemical treatment for the preparation of BaMoO_4_. The obtained structural and luminescent properties will be compared with those of BaMoO_4_ obtained via a solid-state reaction.

## 2. Materials and Methods

### 2.1. Direct Mechanochemical Synthesis

The reagents used in mechanochemical treatment are BaCO_3_ (Merck, purity 99.9%) and MoO_3_ (Merck, purity 99.9%). The stoichiometric ratio of the starting materials was 1:1 and corresponded to crystal-phase BaMoO_4_. High-energy ball milling of the initial mixture was carried out in a planetary ball mill (Fritsch, Premium line, Pulverisette No 7) with two different milling speeds: 500 and 850 rpm. The grinding ball was 2.5 mm in diameter and the ball-to-powder mass ratio was 10:1. Activation was performed in an air atmosphere. To minimize the temperature during milling, the process was carried out for periods of 15 min, with rest periods of 5 min [32,33,34]. The labels of the milled samples were as follows: BaMoO_4_-I using a milling speed of 500 rpm, BaMoO_4_-II using milling speed of 850 rpm.

### 2.2. Solid-State Reaction

The starting materials for the solid-state reaction were the same as those used for the mechanochemical activation, i.e., BaCO_3_ (Merck, 99.99%, Rahway, NJ, USA) and MoO_3_ (Merck, 99.99%). The stoichiometric proportions of both components were mixed and homogenized in an agate mortar at room temperature. Subsequently, the mixture was transferred to an alumina crucible and thermally treated at 900 °C for 15 h in an electrical furnace. The as-prepared sample was marked as BaMoO_4_-III.

### 2.3. Characterizations

Phase identification of BaMoO_4_ was confirmed via X-ray powder diffraction analysis (XRD). The X-ray powder diffraction study was performed using a Bruker D8 Advance instrument (Bruker, Billerica, MA, USA) equipped with a copper tube (CuKα) and a position-sensitive LynxEye detector. The crystallite size and cell parameters were calculated using HighScore plus 4.5 and ReX software (ReX v. 0.9.3 build ID 202308221535 (2023-08-22)). The crystallite size (D) was measured via diffraction line analysis using the Scherrer equation while taking into account the 2θ (angular positions), Int (integral intensity value) and FWHM (full width at half-maximum). LaB_6_ (NIST standard 660c-https://tsapps.nist.gov/srmext/certificates/660c.pdf) was used as a standard [35]. The cell parameters were obtained after refinement of the diffraction lines using the pseudo-Voigt profile function.

Infrared spectra were registered in the range 1200–400 cm^−1^ on a Nicolet-320 FTIR spectrometer using the KBr pellet technique with a spectral resolution of 2 nm. The diffuse reflectance UV-Vis spectra were recorded using a Thermo Evolution 300 UV-Vis Spectrophotometer (Thermo Fisher Scientific, Waltham, MA, USA) equipped with a Praying Mantis device (Harrick Scientific, Pleasantville, NY, USA). For taking background measurements, we used Spectralon. The PL emission spectra were measured using a Horiba Fluorolog 3-22 TCS spectrophotometer (Horiba, Kyoto, Japan) equipped with a 450 W Xenon Lamp (Edinburgh Instruments, Livingston, UK) as the excitation source. This automated modular system has the highest sensitivity among those available on the market, allowing for the measurement of light emission for practically any type of sample. We used double-grating monochromators with emissions in the range of 200–950 nm; Ex. and Em. Bandpasses of 0–15 nm, continuously adjustable from a computer; wavelength accuracy of +/−0.5 nm; and scan speed of 150 nm/s. All spectra were measured at room temperature.

## 3. Results and Discussion

### 3.1. XRD Analysis

**Figure 1:**
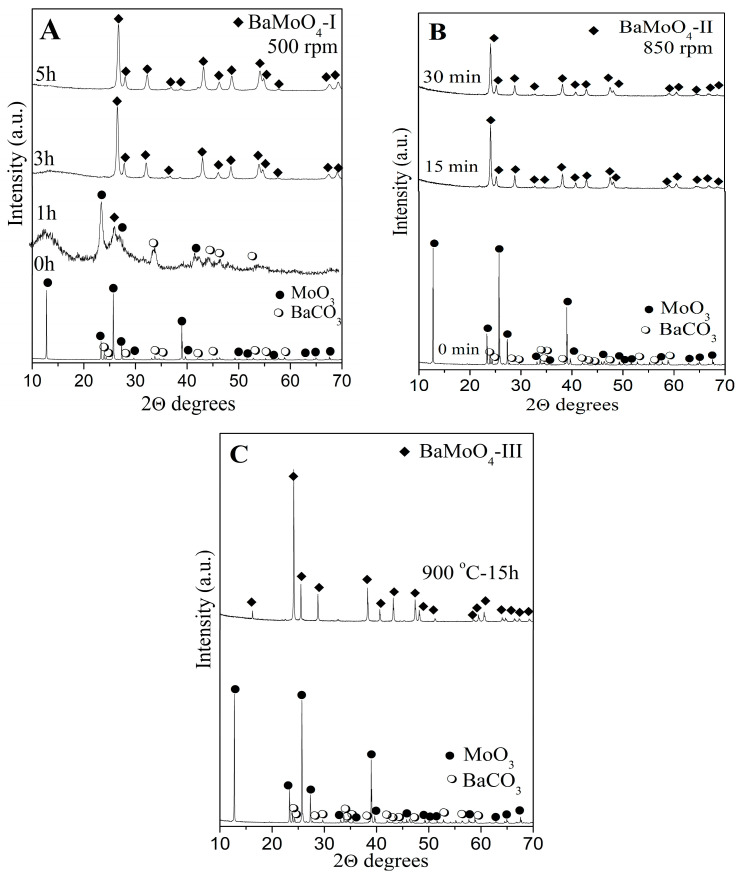
XRD patterns of the initial mixture and mechanochemically activated mixture at 500 rpm (**A**), mechanochemically activated mixture at 850 rpm (**B**) and BaMoO_4_ obtained after solid-state reaction (**C**).

Comparisons of the structure, crystallite size and symmetry of the MoO_4_ units and the optical properties of the samples produced via mechanochemical and solid-state methods were performed. The milling speed is an important parameter during mechanochemical activation, and it was studied. The effects of both milling speeds (500 and 850 rpm) on the reaction time and phase formation of BaMoO_4_ were established via X-ray diffraction analysis (Figure 1A,B). The XRD patterns of the initial mixture before high-energy milling treatment show the principal peaks of orthorhombic MoO_3_ (PDF-98-035-0609) and orthorhombic BaCO_3_ (PDF-98-001-5196). A low milling speed at 500 rpm for 1h led to a decrease in intensity and broadening of the diffraction lines on the initial reagents. This is a result of a decrease in particle sizes, destruction of the long-range order and partial amorphization. In the same X-ray pattern, a new diffraction line at 26.35°, typical for tetragonal BaMoO_4_ (PDF-01-089-4570), was observed. Increasing the milling time up to 3 h caused the appearance of additional reflections characteristic of BaMoO_4_. The small intensity peak at 13.00° and amorphous halo indicate that the full reaction did not occur. The complete reaction between the activated reagents was finished after 5 h of milling time (Figure 1A). In order to verify the reaction time and phase formation of BaMoO_4_, we used a milling speed of 850 rpm. The higher milling speed led to the appearance of the principal peaks of BaMoO_4_ after a shorter time of activation (15 min). But diffraction lines typical of unreacted MoO_3_ were observed at 2θ = 24° (Figure 1B). A single phase of tetragonal BaMoO_4_ was synthesized after 30 min of milling time, which is shorter compared to the reaction time activation was applied at 500 rpm. The phase formation of BaMoO_4_ at the higher milling speed is due to more effective solid-state diffusion between regents during the ball–material collisions. Previously, we pointed out that BaMoO_4_ was obtained via different methods of preparation, which involved additives, solvents and prolonged heat treatment [4,9,13,14,15,16,17,18]. The obtained results demonstrated that activation via high-energy ball milling offers enough energy to generate a chemical reaction at room temperature. It was noted that the ball milling of the mixture of BaCO_3_ and MoO_3_ using the milling speed of 850 rpm produces tetragonal BaMoO_4_ with a faster reaction time. These results demonstrate that mechanochemical treatment is a more substantial approach to the rapid preparation of powder materials. Figure 1C exhibits the XRD pattern of BaMoO_4_ after heat treatment at 900 °C for 15 h. Remarkable narrowing of the diffraction lines was observed, which occurred due to the high crystallinity of BaMoO_4_ compared to the mechanochemically synthesized BaMoO_4_ powders. No additional diffraction lines were found, indicating that the obtained samples were a pure single phase when using both preparation techniques.

Table 1 presents lattice parameters a and c, the volume of the unit cells and the average crystallite sizes for the BaMoO_4_ obtained via different methods of synthesis. A higher milling speed for a short time led to a slight increase in lattice parameters a and c, the volume of the unit cells and the average crystal size. This fact can be attributed to the increase in lattice defects due to higher energy caused by a higher milling speed. A rapid mechanochemical reaction induced a smaller crystallite size due to fast crystallite formation. In solid-state synthesis, the total number of defects significantly decreased, which is demonstrated by an increase in the crystallite size up to 1540 Å. Lattice parameter c decreased compared with those of BaMoO_4_-I and BaMoO_4_-II obtained after a shorter time of synthesis. This can be attributed to the clustering of point defects in the *a* plane, which was also indirectly confirmed via infrared analysis.

### 3.2. Infrared Analysis

**Figure 2:**
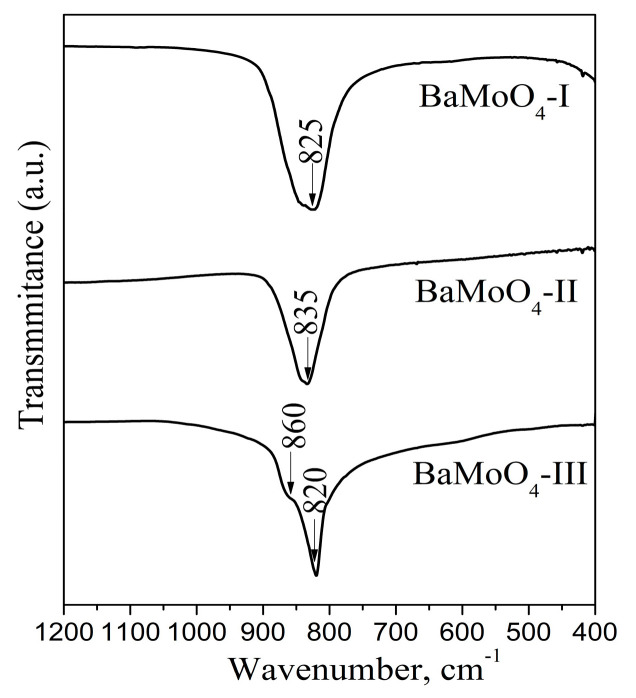
Infrared spectra of BaMoO_4_ synthesized using different methods of preparation.

The phase formation of BaMoO_4_ using different methods of synthesis was confirmed via IR spectroscopy (Figure 2). On the other hand, this analysis is suitable to investigate the local structure of metal ions in the crystallite phases. The infrared spectra display one absorption band in the range of 820 to 835 cm^−1^ due to the *v*_3_ vibration of the MoO_4_ structural units forming the crystalline structure of BaMoO_4_ [1,6,18,24]. The IR spectra of all BaMoO_4_ samples are similar. Small differences in the frequency of the main absorption mode were detected. The band position at 825 is shifted up to 835 cm^−1^ and becomes more symmetric using the higher milling speed of 850 rpm. This fact can be attributed to the formation of more symmetric MoO_4_ units. The partial amorphization and longer milling time at the speed of 500 rpm favors the formation of a distorted MoO_4_ entity. The IR spectrum of BaMoO_4_ prepared via the solid-state reaction exhibits a more narrow and intense absorption band (820 cm^−1^), an indication of the higher crystallinity of sample, which is confirmed via XRD analysis (Figure 1C and Table 1). In this case, the appearance of a shoulder at 860 cm^−1^ is attributed to elimination of the ν_3_ vibration degeneracy of MoO_4_ tetrahedra with different local symmetry [36]. This result can be attributed to the formation of more distorted Mo-tetrahedral groups due to the long sintering time. We can conclude that the mechanochemical activation at higher milling speed leads to the formation of more symmetrical MoO_4_ structural units. Bearing in mind the IR features, we expect that the obtained BaMoO_4_ powders will possess different luminescence behavior according to J.C. Sczancoski et al. [11].

### 3.3. Ultraviolet–Visible Spectroscopy

**Figure 3:**
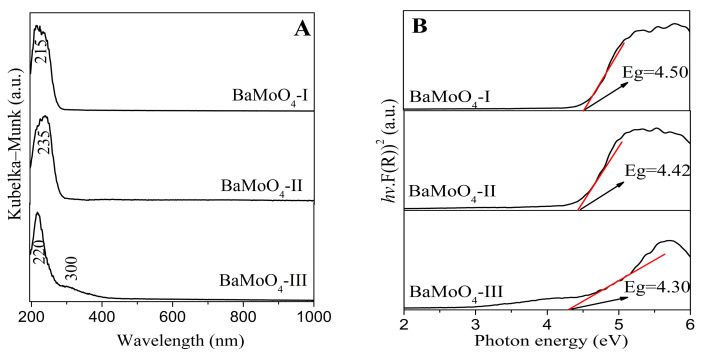
(**A**) UV-Vis spectra of BaMoO_4_ obtained using different methods of synthesis; (**B**) Tauc’s plot of BaMoO_4_ obtained using different methods of synthesis.

Regarding the effects of the band gaps of inorganic materials on their luminescent and electrical properties, according to the literature data, the value of the optical band gap will increase with a decrease in crystallite size [12,37,38]. The UV-vis absorbance spectra of the obtained BaMoO_4_ are shown in Figure 3. All samples exhibit one absorption peak in range 215–235 nm, which is attributed to the charge-transfer transitions within the MoO_4_^2−^ complex [1,3,4,5,6,7,8]. The observed UV-Vis spectra of our samples are closer to those of BaMoO_4_ obtained via the hydrothermal method, the molten salt synthesis and the sonochemical route [4,8,17]. A slight shift in this peak is observed upon applying the different methods of preparation (Figure 3A). In the UV-Vis absorption spectrum of BaMoO_4_-III prepared via the solid-state reaction, a band at 300 nm is also observed. Bearing in mind the reported data, this band corresponds to the creation of the excitonic state in A^2+^ ions (A^2+^ = Ba, Sr, Ca) [3,39]. The optical band gap energy (Eg) of the samples prepared via mechanochemical and solid-state methods was calculated using the Tauc equation [40].

αhν = A (hν − Eg)^n^,

where h is Planck’s constant, α is the absorption coefficient, ν is the photon frequency, A is a constant, Eg is the optical band gap and *n* = 1/2 is used for BaMoO_4_. Based on this equation, the band gaps of the investigated crystal phases are estimated to be above 4.30 eV, as illustrated in Figure 3B. The examined optical band gaps with respect to milling speed and time show a tendency to decrease in value with increasing the crystallite size (BaMoO_4_-I with D = 240 Å and Eg = 4.50 eV; BaMoO_4_-II with D = 270 Å and Eg = 4.47 eV). This tendency was also observed for BaMoO_4_-III (Eg = 4.30 eV with D 1540 Å) prepared via the solid-state reaction. The smaller optical band gap suggests that the crystal structure of BaMoO_4_-III has more defects, which is established via X-ray analysis and IR spectroscopy. The formation of defects led to the induction of additional electronic states between the valence band and conduction band in the materials, resulting in a reduction in the Eg value. The values of the calculated band gaps for milled samples subjected to different speeds are slightly higher than those of samples synthesized via heat treatment at 900 °C. The obtained values of the optical band gaps are higher than those of BaMoO_4_ obtained via hydrothermal synthesis, the coprecipitation method, laser-induced synthesis and the microwave plasma method [4,19,24,41]. According to W. Wang et al., materials with lower crystallite size and with larger values of the optical band gap (Eg) possess higher luminescence intensity [37]. To better understand the correlation between the optical properties and crystal structure, we measured the PL spectra of the obtained BaMoO_4_ samples.

### 3.4. Photoluminescence (PL) Analysis

**Figure 4:**
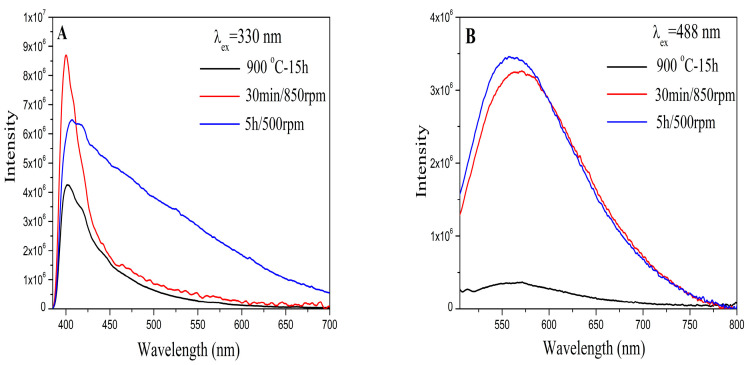
Photoluminescence emission spectra of BaMoO_4_ obtained using different methods of synthesis under excitation at 330 nm (**A**) and 488 nm (**B**).

The photoluminescence emission behavior of the three BaMoO_4_ powders were investigated at room temperature and are presented in Figure 4. An asymmetric and intense blue peak with a maximum at 400 nm was recorded under excitation at 330 nm (Figure 4A). The profile of blue emission lines is similar to those of BaMoO_4_ obtained via the hydrothermal and microwave-induced plasma methods [1,3]. The effect of ball milling speed/time on light emission was investigated and is discussed below. Significant differences were found for the investigated samples. The position of the maximum was changed from 400 to 405 nm for BaMoO_4_-I obtained using the milling speed of 500 rpm. The PL in the blue region was broader after longer milling time at the same rotation speed (BaMoO_4_-I). The lowest intensity was observed for BaMoO_4_-III obtained via the traditional solid-state reaction. A broad green symmetric emission centered at about 560 nm was observed under excitation at 488 nm (Figure 4B). The shape of the PL line is typical of inorganic metal oxide with a scheelite-type structure [20,42]. The PL strength in the green region is almost the same for both samples obtained via mechanochemical activation using milling speeds of 500 and 850 rpm, respectively (Figure 4B). In this case, the broadest peak and lowest intensity were observed for BaMoO_4_-III obtained via the solid-state route. S. Raghunath et al. reported that BaMoO_4_ obtained via the precipitation method possesses lower emission intensity in the range of 510 to 590 nm [43]. According to the literature data, blue emission is attributed to charge-transfer transitions within the [MoO_4_] complex, while the green emission is due to intrinsic distortion in the [MoO_4_] tetrahedron group and can arise due to various factors, like crystallinity, morphology, surface defects, etc. [3,16,20,21,44]. In this instance, the variation in the PL intensity of our samples was caused by the different preparation methods, crystallite sizes and main structural units, i.e., MoO_4_. The above results show that BaMoO_4_-I prepared after a brief milling period using a milling speed of 850 rpm exhibited higher blue luminescence intensity than samples obtained at a lower milling speed of 500 rpm and solid-state reaction, respectively. The broader peak and lower intensity in the blue and green emissions of BaMoO_4_-III prepared via the solid-state reaction are probably due to the presence of more distorted MoO_4_ units according to the obtained IR results. This result is in good agreement with those reported by R. Künzel et al. [4]. The photoluminescence results indicated that the BaMoO_4_ prepared via mechanochemical activation had a small number of defects. The other factor that affected the PL intensity was the crystallite size, which was clearly observed in both emissions. Our investigations show that the BaMoO_4_-I and BaMoO_4_-II obtained via direct mechanochemical synthesis with lower crystallite sizes (240 and 270 Å) possess higher luminescence strength. Similar results have been reported by other authors [37,45]. On the other hand, the lower values of the optical band gap (Eg) and PL emissions of BaMoO_4_ produced via solid-state synthesis were impacted by the formation of distorted MoO_4_ and higher crystallite sizes. From the obtained PL data, we can conclude that symmetric MoO_4_ units and lower crystallite size are important factors for improving luminescence efficiency. Our future investigations will be focused on the mechanochemical synthesis BaMoO_4_ doped with different rare-earth ions (Eu^3+^, Dy^3+^ and Tb^3+^) for obtaining materials with multiple colors. As BaMoO_4_ matrix luminescence is blue or green, the first step will be the preparation of Dy^3+^-doped BaMoO_4_ to achieve the white light emission.

**Figure 5:**
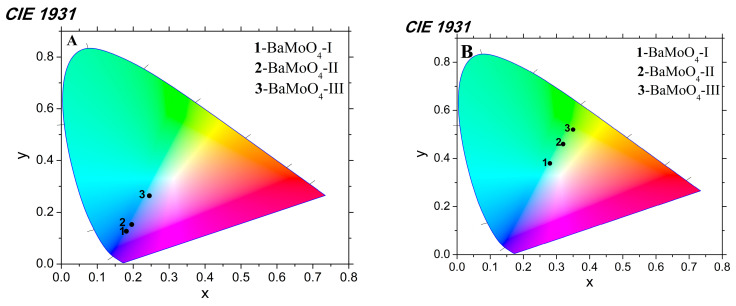
CIE color coordinates of obtained BaMoO_4_ samples under excitation at 330 (**A**) and 488 nm (**B**).

Color chromaticity coordinates are another feature that impacts optical properties. The values of the x and y coordinates of BaMoO_4_ powders were calculated using a standard procedure from their emission spectra and are presented in Figure 5A,B. It can be seen from the figures that they fall within the blue and green areas, respectively. The values of the CIE parameters of the obtained BaMoO_4_ samples are summarized in Table 2.

## 4. Conclusions

In this work we investigate the correlation between the method of synthesis and the structural and optical properties of BaMoO_4_. Nanoparticles of the BaMO_4_ with a scheelite-type structure were prepared via a mechanochemical approach and a solid-state reaction. It was established that the milling speed is a crucial parameter for the rapid synthesis of BaMoO_4_ at room temperature. The crystallite size of both materials obtained at different speeds of mechanochemical activation (500 and 850 rpm) were in the nanoscale range, up to 300 Å. The calculated optical band gaps were wider (above 4.47 eV). The deformation of the structural units that formed the BaMoO_4_ compound was established via IR spectroscopy. It was found that the optical properties depend on the applied method of synthesis, and therefore, on the structural entity distortion and the crystallite size. Mechanochemically prepared BaMoO_4_ had stronger and more symmetric photoluminescence spectra in the blue and green regions compared to the sample prepared via solid-state synthesis. The symmetry of MoO_4_ structural units and crystallite size are both factors that affected the emission intensity. The obtained results suggest the potential use of BaMoO_4_ in the production of optoelectronic devices.

## Figures and Tables

**Table 1:** Lattice parameter values, unit cell volumes and average crystallite sizes of BaMoO_4_ prepared using both methods.

Samples	*a* = *b* (Å)	*c* (Å)	Unit Cell Volume (Å)	Average Crystallite Size (Å)
BaMoO_4_-I 5 h/500 rpm	5.5718 (9)	12.800 (3)	397.3864	240 (5)
BaMoO_4_-II 30 min/850 rpm	5.5811 (4)	12.819 (1)	399.2755	270 (5)
BaMoO_4_-III 900 °C-15 h	5.6083 (2)	12.7019 (5)	399.5213	1540 (4)
PDF-BaMoO_4_	5.58	12.82	399.17	-

**Table 2:** CIE color coordinates of obtained BaMoO_4_ samples under excitation at 330 and 488 nm.

Samples	x, y (exc. 330 nm)	x, y (exc. 488 nm)
BaMoO_4_-I	0.18, 0.12	0.28, 0.38
BaMoO_4_-II	0.20, 0.15	0.32, 0.46
BaMoO_4_-III	0.25, 0.26	0.35, 0.52

## Data Availability

Not applicable.
